# Structural Efficiency of Percolated Landscapes in Flow Networks

**DOI:** 10.1371/journal.pone.0003654

**Published:** 2008-11-05

**Authors:** M. Ángeles Serrano, Paolo De Los Rios

**Affiliations:** 1 IFISC (CSIC-UIB), Instituto de Física Interdisciplinar y Sistemas Complejos, Campus Universitat Illes Balears, Palma de Mallorca, Spain; 2 Institute of Theoretical Physics, LBS, SB, EPFL, Lausanne, Switzerland; University of East Piedmont, Italy

## Abstract

The large-scale structure of complex systems is intimately related to their functionality and evolution. In particular, global transport processes in flow networks rely on the presence of directed pathways from input to output nodes and edges, which organize in macroscopic connected components. However, the precise relation between such structures and functional or evolutionary aspects remains to be understood. Here, we investigate which are the constraints that the global structure of directed networks imposes on transport phenomena. We define quantitatively under minimal assumptions the structural efficiency of networks to determine how robust communication between the core and the peripheral components through interface edges could be. Furthermore, we assess that optimal topologies in terms of access to the core should look like “hairy balls” so to minimize bottleneck effects and the sensitivity to failures. We illustrate our investigation with the analysis of three real networks with very different purposes and shaped by very different dynamics and time-scales–the Internet customer-provider set of relationships, the nervous system of the worm *Caenorhabditis elegans,* and the metabolism of the bacterium *Escherichia coli.* Our findings prove that different global connectivity structures result in different levels of structural efficiency. In particular, biological networks seem to be close to the optimal layout.

## Introduction

Despite profound differences, natural and artificial networked systems share striking similarities. Complex networks science [1], [2], [3] has successfully identified several of their common features, such as the small world property or the presence of strong degree heterogeneity, relating them to the existence of organizing principles. These ubiquitous properties seem further reinforced by the universality of patterns recognized in the large-scale architecture of a class of networks describing transport processes and represented as directed networks. They are characterized by asymmetric interactions giving rise to local flows–of matter, energy, information, etc.–that collectively organize into a global stream dominated by three main structures, an input component and an output one connected by a core. The precise relation of these characteristic bow-tie layouts [4] to functional or evolutionary aspects remains to be understood and prompts for an in-depth investigation.

In general terms, previous research exploring the relation between form and function in complex networks has mainly focused on the analysis of topological features such as modular ordering related to functional aspects [5], with only a few exceptions treating directly concepts such as efficiency [6]. Specifically, transport has been studied as one of the main functions influenced by topology [7], [8] and functional design principles of global flux distributions have been discussed for biological networks [9], [10], [11]. Despite these efforts, the “form follows function” assertion still remains to be fully understood from a complex network science perspective, a major difficulty being the fact that present network patterns are the result of non-stationary and adaptive evolutionary histories that can greatly vary depending on the network. Such interplay between structure formation and evolution is usually studied by modeling networks where connections change depending on the node dynamics [12], [13], [14], [15]. In some of these models [16], spontaneous structures are able to form that exhibit the typical architectures of transport systems.

Our purpose of inferring information about function and evolution from a precise knowledge of the topology requires the understanding of how global transport networks organize to develop functionality. In this respect, percolation theory of complex networks [17] provides a valuable framework to characterize the presence and the size of the connectivity structures that are essential for the description of networks at the macroscopic level. Their global connectivity layouts in the percolated phase are named henceforth as percolated landscape. The analysis of these structures allows us to quantify the degree of efficiency that networks are able to achieve as global transport systems. In particular, the major role played by the interfaces bridging the different percolated components [18] allows us to discuss how accessible the core is for elements in the peripheral components. We define structural efficiency in terms of the stress (or load) carried by the interfaces as elements transported in the system traverse the network. This quantity also gives information about robustness, indicating the edges at the interfaces that are more critical in connecting the periphery to the core. Finally, we use theoretical arguments to propose the conformation of maximal structural efficiency. Although our aim is not to perform a detailed comparative analysis, we illustrate our investigation with the examination of real networks with very different purposes. Their analysis allows us to show that different percolated landscapes impose different structural restrictions on transport and as a consequence networks display different levels of structural efficiency. In particular, we studied two biological systems exposed to long-term evolutionary pressures, which seem to be close to optimality, and an information technology system such as the Internet, at an early stage of development and dominated by competitive forces, which is far from an efficient global architecture.

## Results

### Percolated landscapes of directed networks

**Figure 1:**
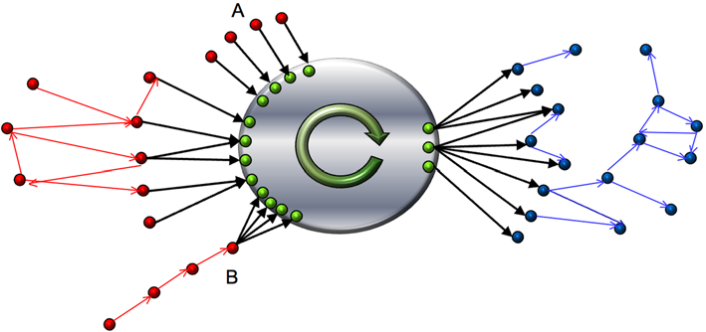
Schematic diagram of the main components in the percolated landscape of a flow network. The core at the center comprises nodes in the strongly connected component (SCC) and edges within (SCE). The rest corresponds to peripheral components. Nodes in red belong to the in node component (IN) and the edge in component (ICE) is formed by red links. Nodes in blue belong to the out node component (OUT) and blue links form the edge out component (OCE). Both interfaces (ITF and OTF) appear in black.

For all complex systems, global communication is essential to develop efficiently collective behavior. In directed network representations, it is ensured by the presence of pathways that enable to pass from the input to the output components. These global layouts are best described in the framework of percolation theory [17]. Above the percolation transition, the topological landscape denotes a global flux that organizes in different linked components comprising macroscopic portions of the system (Fig. 1 gives a schematic representation). The node percolated map [19], [20], [21], [22] organizes around a core structure, the strongly connected component (SCC), whose vertices can communicate with each other following directed paths. This core is usually connected to peripheral components: the in-component (IN), composed of all vertices that can reach the SCC but cannot be reached from it, and the out-component (OUT), made of all vertices that are reachable from the SCC but cannot reach it. Secondary structures, such as tubes or tendrils, could also be present [4]. Changing perspective from nodes to edges, this picture is complemented by the edge percolated map [18], where the number of relevant structures increases to five: edges connecting nodes within the IN, OUT, and SCC form respectively the edge in component (ICE), the edge out component (OCE), and the edge strongly connected component (SCE); and edges bridging the peripheral IN and OUT components to the SCC form the in and the out interfaces (ITF and OTF respectively). Edges at the interfaces connect nodes in different components so that they have a hybrid nature that prevents to classify them in a single node component. The edge interfaces are thus essential and there is no transformation able to reduce the edge percolated map to the node percolation map (in contrast to the duality between nodes and edges in undirected network representations). They both are complementary and indispensable for a complete description of the large-scale structure of percolated networks.

The global organization of directed networks is further shaped by system dependent specificities that are the reflection of functional demands and evolutionary and/or adaptive forces. In particular, the specific conformation of the interfaces informs us about the potential risk of bottleneck effects and more in general tells us about how well structure facilitates global communication in the system.

### Real systems

We consider here three different systems characterized by global transport phenomena: a socio-technological one governed by competitive forces, namely the set of customer-provider relationships between autonomous systems in the Internet, and two natural cooperative systems, the nervous wiring of the nematode worm *Caenorhabditis elegans* (*C. elegans*) and the metabolism of the bacterium *Escherichia coli* (*E. coli*). Our aim is not to perform an exhaustive comparative analysis but to prove that different real systems could display very different percolated landscapes and as a consequence very different levels of structural efficiency.

The Internet is one of the paradigms of information and communication networks [23]. From an operative point of view, it is composed of thousands of autonomous systems that operate individual parts of the whole infrastructure. Those engage in mutual business relationships [24] to collectively route traffic through the network, giving place to transfers of money in a competitive market. Such dependencies can be mapped to a directed graph representation of unambiguous customer-provider relationships (see Materials and Methods).

A different family of archetypical information transport systems, dominated by cooperative rather than competitive forces, are biological nervous systems. As for human-made complex networks, their structure is intimately related to their function and they display an emergent behavior that cannot be understood as being merely the sum of the individual actions [25]. As a particular example, here we focus on the nervous system of the worm *C. elegans* which is practically completely known [26], [27], and reconstruct its synaptic structure as a directed network (see Materials and Methods and Supporting Information Table S3).

Cellular metabolism is also recognized as a canonical biological transport system and its bow-tie structure [10] has been recognized to be compatible with the categorization of metabolites into a variety of nutrient inputs that are transformed into intermediate metabolites necessary to the biosynthesis of final compounds. We have thus chosen as a third example the network representation of the metabolism of the bacterium *E. coli* [28], [29] (see Materials and Methods for its network reconstruction).

**Figure 2:**
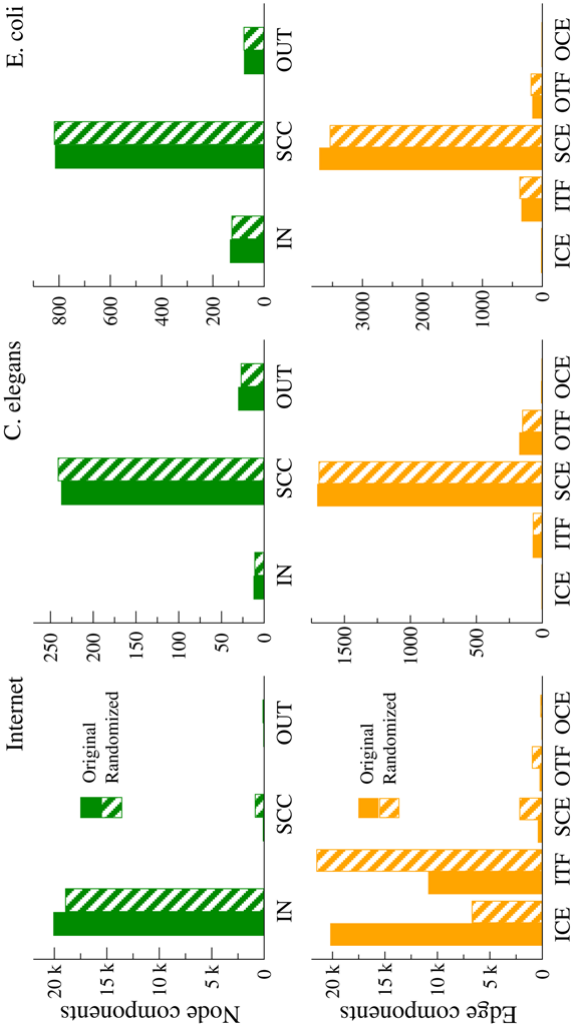
Bar charts detailing the percolated landscapes of the real network. Values for the Internet customer-provider relationships network, the synaptic neuronal network of *C. Elegans,* and the metabolism of *E. coli* are compared with their randomized counterparts. Top charts show the node components and the bottom charts show the edge components. The total number of nodes and edges are 24545 nodes and 45914 edges for the Internet, 279 nodes and 1961 links (of which 233 are bidirectional) for *C. elegans,* and 1024 nodes and 4283 links (of which 634 are bidirectional) for *E. coli*. See Table S1 in Supporting Information for detailed numerical values.

These networks present different global structures. Their node and edge percolated maps are shown in Fig. 2 (see also Table S1 in Supporting Information) as compared to their maximally random counterparts analyzed as null models (see Materials and Methods). The node percolated map of the Internet customer-provider network is extremely asymmetric and hierarchical, with a huge IN component, a very small SCC, and an even tinier OUT component, in perfect accordance to CAIDA's AS rank classification based on the size of the customer cones for the same dataset [30]. Even though the SCC comprises less than one percent of the total number of nodes in the network, its presence implies that the Economic Internet is not a perfect acyclic graph but contains a small number of directed loops. While at first glance this may seem counterintuitive, we remember that we are analyzing customer-provider relationships and not traffic flows and the presence of structural loops in economic networks is common [31]. Finally, we note that the randomized counterpart is qualitatively very similar. On the other hand, the edge percolated map shows that the architecture of the real network is different from that of the randomized one. While the number of edges in the core and the outgoing structures are qualitatively in accordance with the values for the randomized network, the organization of the incoming components is very different from random. The ICE of the real network contains as many edges as nodes in the IN component, which points to a tree-like structure. Moreover, the number of ITF edges connecting the IN and the SCC is just half the number of IN nodes so that many nodes in the in-component lack direct access to the core as expected in a hierarchical system. In contrast, the randomization produces an ITF with twice as many edges than actually observed, with a correspondingly reduced ICE, so a shallower IN component.

Unlike the Internet, the percolated layouts of the synaptic neuronal structure of *C. elegans* and the metabolism of *E. coli* are surprisingly close to a random organization (once preserved the number of connections per node). In both cases, the main structure consists of a big core with smaller IN and OUT components in accordance to the measures for the randomized versions. The number of edges within the peripheral components is extremely small. As a result, the *C. elegans* nervous system seems to rely on clear input and output signals with direct access to the SCC, the computational processing core, through well populated interfaces. For the metabolic network, the organization of edges reveals that almost all reactions occur in the core, with input nutrients directly entering it without pre-processing and biosynthesized compounds leaving it in its final form.

### Structural efficiency in terms of stress

Next, we show how these conformations may impose bounds to performance assuming that the basic functionality of a flow network is to perform a global transport. Interfaces play the pivotal role of connecting core and periphery and, setting aside the discussion of weights [32] (see Materials and Methods) or wiring costs [33], their potential efficiency in fulfilling such task may be defined as a first approximation using structural measures. We introduce two measures of structural efficiency giving idea of how efficiently peripheral elements access the core. More specifically, we define stress as the amount of load that the edges at the interface bear. In this way, we try to characterize with simple measures and under minimal assumptions how robust communication between core and periphery could be and which are the bounds imposed by the structure on global transport.

#### Stress as random-walk betweenness

Thinking of interfaces as bridges, the average structural efficiency of an interface can be simply approximated as
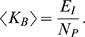
(1)
*E_I_* refers to the average number of edges at the interface *I* (ITF or OTF) and *N_P_* to the number of nodes in the corresponding peripheral component *P* (IN or OUT). High values of 〈*k_B_*〉 are clearly desirable as peripheral nodes would have, on average, more edges -and so more routes- to access the core.

A more detailed description beyond 〈*k_B_*〉 can be achieved by calculating the distribution of loads of the edges at the interfaces. Load can be understood as a measure of the extent to which edges are under stress because of the flow passing through them. It can be characterized as betweenness, a topological measure of the number of paths between nodes in different components that traverse an edge, as elements transported in the system travel the network. Typically, betweenness is calculated taking into account only shortest-paths between pairs of nodes [34]. Here, we are however interested in a basic approximation so we assume that the flow uses only local information without global knowledge of the system. We suppose that the topological structure is supporting blind flow, and we measure the load as random-walk betweenness [35] that counts all possible routes assuming that information wanders at random until it finds the target (see Materials and Methods for further details and the explanation of computational procedures). Edges with higher random-walk betweenness are expected to be more important for the spread of information across the system and, if the load is excessive, bottleneck effects could even appear. Finally, notice that the inverse of the average betweenness of the edges at the interfaces
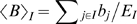
(2)coincides with the structural efficiency average degree defined in Eq. (1). This formalism can be easily extrapolated to situations in which the flow follows other routing protocols.

**Figure 3:**
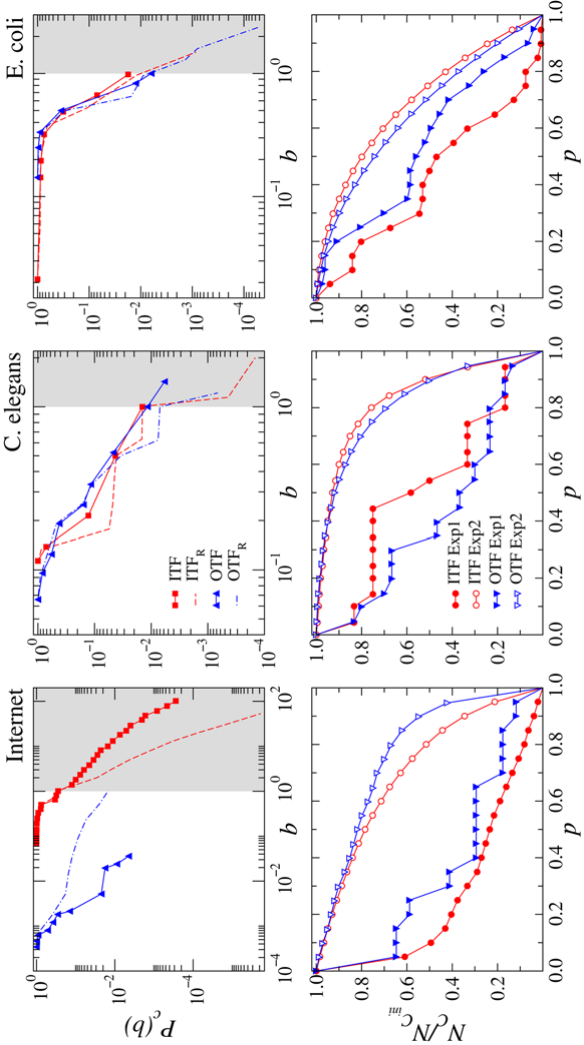
Top plots. Cumulative random-walk betweenness distribution for the in- and out-interfaces. The empirical distributions (symbol lines) are compared against those for the randomized null models (dashed lines). Grey areas denote stress regions with loads above 1. The steps observed for the distributions of the randomized version of the *C. elegans* network (more important for its ITF) are due to the small size of the peripheral component (only 12 nodes in the case of the IN) along to its structure with all nodes directly connected to the core though multiple edges. The small size of the peripheral component brings out the quantization in the number of connections per node at the interface, and this affects the distribution of loads. This structural effect, determined by the degree distribution, cannot be smoothed by the randomization procedure. Bottom plots. Fraction of nodes remaining in the peripheral components after removing a fraction p of edges of the corresponding interfaces. Two different experiments are performed and compared with each other, always on the real networks. In Experiment 1 (full symbol lines), a targeted removal of edges at the interfaces is performed in decreasing order of load as measured in the original network. In Experiment 2 (open symbol lines), the order of edge deletion at the interfaces is random and also applied to the original networks under analysis. In this case, averages are shown over 100 different realizations of random orderings of the edges.

In Fig. 3 (top plots), we provide the cumulative distribution 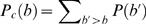 of the random-walk betweenness, or loads, of the edges at the interfaces of the Internet, *C. elegans,* and *E. coli* networks. The graphs show that the loads are not uniformly distributed for either network but are broadly distributed denoting large fluctuations, with a few links bearing a much higher level of structural stress. This heterogeneity is not *per se* indicating that the interface is overstressed. The random-walk betweenness is moderately highly correlated with degree [35] meaning that, in general, edges attached to vertices with high degree tend also to have high random-walk betweenness, so that strong disorder in the topology could induce spurious heterogeneity in the load distribution.

In order to assess whether the structural load could represent a potential danger of bottleneck formation in traffic related processes running on the network, one has to further define what is expected as a low load in the situation of maximal structural efficiency. We make the assumption that such efficiency is reached whenever each edge at the interface carries at most a unitary load. This gives a simple criterion which enables to compare different networks but also different links of the same interface. At the same time, the results should again be validated by the investigation of the maximally random version of the network. In Fig. 3 (top plots), grey areas denote stress regions with loads above 1. Although the distributions for the *C. elegans* and the *E. coli* networks present different functional form, both are once again in good agreement with their randomized versions having almost all loads below the threshold, and remaining quite symmetric in relation to the performance of the ITF and OTF. However, the Internet interfaces are clearly asymmetric. Most edges of the in-interface lie in the grey region, a signature of overstress, and the loads in the real network are above the ones in the randomized network, pointing to a vulnerability of the system. On the contrary, the loads of the out-interface are well below one and below the randomized. For all networks, the region of loads much below one usually corresponds to peripheral leaf nodes directly connected to the core through multiple interface edges. Apart from a signature of local robustness, such diversification could also be interpreted as peripheral leaf nodes being spreaders, if they belong to the IN component, or collectors, if they belong to the OUT. It is noteworthy that the steps observed for the distributions of the randomized version of the *C. elegans* network (more important for its ITF) are precisely due to the presence of these structures. When the size of the peripheral component is very small (only 12 nodes in the case of the *C. elegans* IN, see Table S1 in Supporting Information), the quantization of the number of connections associated to the peripheral nodes at the interfaces becomes noticeable and affects the distribution of loads. This structural effect cannot be smoothed by the randomization procedure because it preserves the degree distribution.

The distribution of loads also informs us about robustness, defined as a measure of the ability of the interfaces to maintain communication between core and periphery under malfunction or failure. In the bottom plots of Fig. 3, we show the fraction of nodes in the peripheral components that remains connected after the removal of an increasing fraction of interface edges. Two different experiments are performed, both of them on the real networks. The first removes edges in decreasing order of load and the second selects at random the edges to be removed at the interfaces of the original network. The results prove that although the interfaces seem to be quite robust against random failures, the malfunction of high load edges would disconnect a bigger portion of peripheral nodes, strongly affecting the behavior of the system. This effect is more important in the case of the Internet, for which a sharp drop of about 40% is observed in the number of connected nodes for p<0.1. This is not found in the curves for the other two networks with loads which are not as broadly distributed. Again, steps appear in the curves for the Internet OTF and the *C. elegans* interfaces (more evident for its ITF). The reason is, as before, the presence of spreaders and collectors in small peripheral components. Their edges at the interfaces share equal load, so that when removing edges by load those peripheral nodes do not become disconnected until all its edges disappear, giving place to the observed small plateaus. The metabolic network is characterized by a distribution of loads far from homogeneous but nevertheless narrower than the ones associated to the other two networks. As a consequence, it does not seem to be as sensitive as them regarding the targeted removal of edges by load. In particular, the curves for the two experiments performed on the *E. coli* OTF are quite similar, except for a sharp drop in the curve for the targeted experiment in the range 0.15<p<0.35.

#### Discounting leaf edges at the interfaces

The random walk methodology presented above cannot discriminate between peripheral conformations with different access to the core if equal loads are associated to their edges at the interface (as a simple example, see tree-like groups A and B in Fig. 1). A refinement is needed to characterize the different efficiencies associated to dissimilar peripheral architectures.

By convention, leaf vertices are those with in-degree zero or out-degree zero, so that they are restricted to belong to a peripheral component (if present, bidirectional links have to be counted as an incoming and an outgoing link, and by definition can never belong to an interface). In-leaf edges (out-leaf edges) are considered as directed links leaving from (pointing to) a leaf vertex. Strictly speaking, vertices with in-degree zero are usually referred as root vertices. We refer to them as leaf vertices for economy of language. Note also that according to the definitions of in-leaf (out-leaf) edges, the in (out) interface cannot contain out-leaf (in-leaf) edges. From the perspective of vertices the definitions would be reversed.

Non-leaf edges at the interfaces are the ones that ensure the communication from/to nodes not directly connected to the core. These non-leaf edges are the ones potentially responsible for bottleneck effects, since they act as bridges for more than a single IN or OUT node. A first estimate of how these topological considerations affect efficiency at the structural level is given by the average degree,
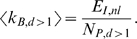
(3)This is the ratio of the number of interface non-leaf edges, *E_I_*_,*nl*_, to the number of peripheral nodes which are not directly connected to the SCC but at a distance d from the core greater than one, *N_P_*_,*d*>1_. (See Table S2 in Supporting Information for the figures associated to the decomposition of the Internet, *C. elegans* and *E. coli* interfaces and peripheral components into leafs and non-leaf units).

Values for the average degree efficiency measures as defined in Eq. (1) and Eq. (3) are shown in Table 1. In general terms, the higher the averages the more structurally efficient the system is expected to be. An important imbalance is observed between the in and out values for the Internet. According to these numbers, the in-interface presents a certain level of inefficiency, with low average degrees combined with a low number of leafs, much below random expectations. In this situation, potential bottleneck effects are more likely. In contrast, the out component shows high levels of structural efficiency, with the practical totality of nodes being directly connected to the core. On the other hand, all peripheral nodes (except a 5% in the *E. coli* IN) of the biological networks analyzed here have direct access to the core, a signature of high efficiency.

**Table 1:** Structural efficiency average degrees.

AVERAGES	Internet	Internet randomized	C. elegans	C.elegans randomized	E. coli	E. coli randomized
〈k_B_〉_IN_	0.54	1.14±0.09	5.83	6.3–0.8	2.60	2.98±0.13
〈k_B,d>1_〉_IN_	0.24	1.05±0.09	∞	∞	4.57	∞
〈k_B_〉_OUT_	13.29	7.9±1.6	5.73	5.6–0.3	2.08	2.39±0.09
〈k_B,d>1_〉_OUT_	8	22±17	∞	∞	∞	∞

Structural efficiency average degrees as defined in Eq. (1) and Eq. (3) for the Internet customer-provider relationships network, the synaptic neuronal network of *C. elegans,* and the metabolism of the bacterium *E. coli*, and their randomized counterparts (values are average±standard deviation rounded off to the first significant figure). Infinite values come from the fact that all peripheral nodes are directly connected to the core.

## Discussion

Under the requirement of low stress, and in the approximation of inexpensive homogeneous edges, maximum efficiency would be realized by a percolated landscape structured as a perfect “hairy ball”, with all the nodes in the peripheral components directly attached to the core through leaf edges carrying at most a unitary load, thus not prone to bottleneck effects. The absence of nodes at distances larger than one could however involve a marginal deviation from the “hairy ball” conformation, with a few loads slightly greater than one due to inner connections in the peripheral component. In the hairy ball layout, the interfaces would be robust because the failure or malfunctioning of any of its edges would affect a minimum number of nodes in the peripheral components. Finally, all peripheral nodes would have direct access to the core. Any departure from the “hairy ball” paradigm would lead to situations in which the requests for structural efficiency as defined here are violated to some extent.

Hence, our analysis of percolated landscapes shows that the conformation of interfaces plays a central role in the performance of complex directed networks representing global transport systems, affecting their efficiency against bottlenecks and their robustness against failures. We emphasize that a “hairy ball” design would be optimal from the point of view of structural efficiency as defined here. As seen for the synaptic neuronal network of *C. elegans* or the metabolism of *E. coli,* such behavior may be displayed even by architectures in good agreement to their randomized counterparts, meaning that their large-scale structures do not need to conform to special ordering requirements beyond the degree distribution. In contrast, the Economic Internet presents a downgraded in-interface, indicating a structural inefficiency. These findings point to two, not mutually exclusive, interpretations. On the one hand, different adaptation dynamics are surely at work: whereas the present structure of the biological networks seem to optimize the collective performance without apparent internal competition pressures, the Internet network emerges, due to its underlying business implications, as a competitive system where it is not the global optimization which is sought for but rather the individual Internet provider gain. In this respect, global efficiency in the sense defined in this work is important because it is necessary as a substrate for the services that Internet providers offer, but it is not a common good that everybody strives to optimize cooperatively. On the other hand, the evolution of the worm nervous system and of the metabolism of the bacterium might have allowed more efficient architectures to emerge, due to the evolutionary time-scales (hundreds of millions of years or more) running much longer than the time-span of existence of the commercial Internet.

Clearly, these findings only shed light on some basic structural ingredients for efficiency and robustness, but this study forms a baseline for trying to discover what other relevant factors are at play. Indeed, several other constraints, *e.g.* costs of edge deployment and maintenance, capacities, weights (see Materials and Methods), or different routing strategies should be taken into account for more precise and system specific analysis. Yet, the structure of a percolated landscape puts fundamental bounds on performance, possibly suggesting specific actions to reinforce stressed elements or to redistribute loads so to reduce the risk of bottlenecks and the impacts of failures.

## Materials and Methods

### Directed network reconstructions

#### Internet customer-provider relationships

The directed graph is reconstructed from the map 2007-04-02 of inferred autonomous systems relationships provided by CAIDA [30]. Relationships among autonomous systems are usually in the form of business agreements, generally simplified to customer-provider, peer-to-peer and sibling-to-sibling. In a purely directed version of the network, where links represent net flow of payments for services provided, relations between siblings immediately cancel out since they administratively belong to the same organization. Peer-to-peer relations are however not trivial because they just freely exchange traffic between themselves and their customers but not up in the hierarchy. Anyway, we assume here that the later are balanced in both directions so as a first approximation we neglect them as well. On the other hand, customer-provider relationships are unambiguously represented by directed edges from customer to provider. We are left with a purely directed network of 24545 nodes and 45914 directed links, after removing 4312 (8.55%) peer-to-peer and 236 (0.47%) sibling-to-sibling relations (nevertheless, we have checked that the consideration of these links as bidirectionals does not alter qualitatively the results). The in-degree distribution is very broad and well described by a power law with characteristic exponent 2.1. The out-degree distribution is strongly bounded and decays extremely fast with a maximum out degree of 24.

#### Synaptic neuronal structure of *Caenorhabditis elegans*

Network representations of brains display neurons as vertices and connection between pairs are present whenever a synapse or gap junction has been observed. We use the updated data set [27] compiled for the analysis of the relation between neuronal placement and wiring costs. [26] The pharyngeal system comprises 20 neurons and is almost totally disconnected from the rest of the network. It is excluded along unconnected neurons, as well as connections of the somatic nervous system to non-neural cells. We further restrict to chemical synapses excluding gap junctions, very different from the previous in nature and function. For simplicity, the polarity and the multiplicity of the connections are not taken into account but directionality is. Most synapses are directed in nature, but 233 reciprocal connections have been detected out of a total number of 1961. We take them into account as bidirectional connections. The network also contains 279 neurons as nodes. As reported previously, it turns out to be a small-world network [36] with tails of the cumulative distribution of degrees for both incoming and outgoing neuronal links that have been reported to be well approximated by exponential decays [37]. See Supporting Information Table S3 for validation of our semidirected network reconstruction in relation to neurons functional types.

#### Metabolism of *Escherichia coli*

Metabolism admits a natural bipartite network representation where metabolites and the biochemical reactions they take part in are represented as two different classes of nodes, linked whenever one metabolite participates in one reaction. It is however more convenient to work with the one mode projection restricted to node metabolites, where substrates in a reaction are connected to products [38]. It has been argued that it is more meaningful to restrict to the most relevant biochemical transformations in every reaction and remove carrier metabolites such as water, ADP, or ATP [39], [40]. Depending on the purpose of the investigation, it is however important to keep all the reactants. In our study, we are interested in the global organization of the connectivity structure of the system so that all elements providing connectivity are relevant. We keep all metabolites as nodes and connect all substrates in a reaction to all products. Irreversible reactions give place to directed links from substrates to products, and reversible reactions generate instead bidirectional links, so that the reversibility information of the bioreactions is encoded in the network. As a representative token, we use the *in silico* reconstruction of the bacterium *E. coli* [28] compiled by the Systems Biology Research Group at UCSD available at the BiGG database [29] (other metabolic networks will be extensively analyzed elsewhere). The list of metabolic reactions of this organism has been curated so to clean it free of transported reactants just carried from the extracellular media to the periplasm or from it to the cytoplasm–or viceversa- without suffering any transformation, that would have produced self-loops in the final network representation. It contains 1024 metabolites as nodes and 4283 links, of which 634 are bidirectional.

### Randomization as a null model

The appropriate null model to find opportune baselines for the detection of anomalies in percolated landscapes corresponds to the maximally random realization of the networks that preserves the degrees of the nodes. For purely directed networks without bidirectional links–such as the Internet customer-provider relationships network-, it is achieved at the stationary state of a rewiring process that at each time step randomly selects a couple of links and exchange their ending points [41] avoiding the formation of multiple and self-connections and bidirectional links. In this way, the incoming degree and the outgoing degree of nodes is preserved.

If bidirectional links are present–as for the semidirected network representations of the nervous system of *C. elegans* and of the metabolism of *E. coli-,* directed and bidirectional links should be treated separately. Following the methodology used in [42], the rewiring algorithm described in the previous paragraph is applied just to the subset of directed links present in the network, and complemented with the usual rewiring procedure in undirected networks applied independently to the subset of bidirectional links, avoiding equally the formation of multiple and self-connections. The randomized networks preserve in this form the in-degree, the out-degree, and the bidirectional degree of the nodes.

Notice that these rewiring processes are not able to destroy structural degree–degree correlations which correspond to constraints ensuring the realizability of the network [43] and are so unavoidable. The comparison of real networks with their randomized counterparts makes therefore possible to determine to which extent the measured values are due to global organizing principles and not to random assemblages affected by finite-size effects. In this work, the randomized versions are produced out of 100 randomized realizations.

### Calculation of loads as random-walk betweenness

In order to calculate the random-walk betweenness of the edges at the interfaces, we slightly modify the original proposal of a centrality measure for vertices [34]. The percolated landscape is explored by means of two symmetric random walks on the unweighted directed network with homogeneous diffusion probabilities and absorbing sinks in the nodes of the SCC. Nodes in the IN act as sources of diffusive particles -either units of energy, packets of information, economic goods, monetary units, etc.–which spread from neighbor to neighbor following outgoing links (also bidirectional if they are present), each chosen with equal probability among the possibilities. The hopping process is stopped whenever the diffusive particle arrives to a node in the SCC following a given link in the ITF, which receives the annotation. The symmetric process originates particles in the nodes of the OUT, which travel backwards following incoming links (also bidirectional edges if present) selected with equal probability among the possibilities, and the diffusion is equally stopped whenever a node in the SCC is reached through a particular link in the OTF, which receives the annotation. By repeating the processes a sufficient number of times for each source node, it is possible to obtain the probability vector that a traveling unit originated at one of the peripheral components uses the edge j in the corresponding interface to reach the core. After multiplying by the size of the source component in number of nodes *N_P_*, the resulting vector 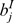 informs about the structural load that each link in the interface supports. Vector 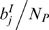 corresponds to a normalized probability distribution whenever tendrils or tubes are not considered. Those appendices act as *cul-de-sac* which receive part of the diffusion unloading partially the interfaces.

### Structural efficiency of weighted networks

The definitions for the structural efficiency average degrees can be easily extrapolated to the case of weighted networks [31], where the intensities of the interactions are also considered as weights. The structural efficiency average weights per node can be expressed as
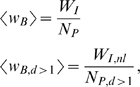
where *W_I_* in the first average represents the sum of the weights on the edges at the interface and the normalization is again the number of corresponding peripheral nodes, while *W_I,nl_* in the second average represents the sum of the weights on the non-leaf edges at the interface and the normalization corresponds to the number of peripheral nodes at distances of the core larger than 1.

In the same way, the loads of single edges at the interfaces can also be calculated as a random walk-betweenness taking into account weights. It would be necessary to modify the exploratory random walks so that the diffusion probabilities are proportional to the weights of the edges selected to expand the process.

## Supporting Information

Table S1 Statistics of the Internet, C. elegans, and E. coli node and edge network components. Values of the sizes of the different node and edge components of the Internet customer-provider network of relationships at the autonomous system level, the nervous system of the worm C. elegans, and the metabolism of the bacterium E. coli, along the values for their randomized counterparts (values are average standard deviation rounded off to the first significant figure). The sizes of the main components are given in absolute number of nodes and links. When present, bidirectional links are counted as single. See Fig. 2 in the main text for a graphical representation.
(0.04 MB DOC)

Table S2 Details of the interfaces and peripheral components of the Internet, C. elegans, and E. coli network representations. Decomposition of the interfaces and the peripheral node components. Values for the randomized versions of the networks are also provided (average standard deviation rounded off to the first significant figure). Edges at the interfaces and nodes at the components are separated into leafs (l) and non-leafs (nl), and nodes directly connected to the core (d = 1) are distinguished from those at larger distances (d>1). When present, bidirectional links are counted as single.
(0.04 MB DOC)

Table S3 Distribution of C. elegans neurons by function type among the different node components. To check that our production of the semidirected network is consistent and meaningful we computed the distribution of neurons by function type among the different node components. C. elegans neuronal functional organization is similar to most other metazoans and basically consists of sensory neurons (se), interneurons (in), and motor neurons (mo). We follow the classification in [1], which separates these cells into the three main groups and their crossings. Our statistical analysis reveals a structured overlap between the node components and the functional organization of neurons by type. Interestingly and according to rational expectations, the IN is mainly formed by sensory neurons while the OUT primarily consists of motor neurons, apart from interneurons present in both. Neurons in the SCC span all the classes with similar proportions.
(0.03 MB DOC)
