# Supplementary material for: Structural Efficiency of Percolated Landscapes in Flow Networks
Source: PLoS One. 2008 Nov 5;3(11):e3654. doi: 10.1371/journal.pone.0003654 (PMC2575234; doi:10.1371/journal.pone.0003654)
Supplement: Table S1 — Statistics of the Internet, C. elegans, and E. coli node and edge network components. Values of the sizes of the different node and edge components of the Internet customer-provider network of relationships at the autonomous system level, the nervous system of the worm C. elegans, and the metabolism of the bacterium E. coli, along the values for their randomized counterparts (values are average standard deviation rounded off to the first significant figure). The sizes of the main components are given in absolute number of nodes and links. When present, bidirectional links are counted as single. See Fig. 2 in the main text for a graphical representation. (0.04 MB DOC) [file pone.0003654.s001.doc]

## TABLE S1. Statistics of the Internet, *C. elegans*, and *E. coli* node and edge network components

Values of the sizes of the different node and edge components of the Internet customer-provider network of relationships at the autonomous system level, the nervous system of the worm *C. elegans*, and the metabolism of the bacterium *E. coli*, along the values for their randomized counterparts (values are average$\pm$standard deviation rounded off to the first significant figure). The sizes of the main components are given in absolute number of nodes and links. When present, bidirectional links are counted as single. See Fig. 2 in the main text for a graphical representation.

| **NODE COMPONENTS** | **Internet** | **Internet randomized** | ***C. elegans*** | ***C.elegans* randomized** | ***E. coli*** | ***E. coli* randomized** |
| --- | --- | --- | --- | --- | --- | --- |
| **IN** | 20060 | 18900±700 | 12 | 11±1 | 132 | 126±4 |
| **SCC** | 90 | 850±40 | 237 | 240.7±1.2 | 814 | 818±4 |
| **OUT** | 17 | 120±20 | 30 | 27±1 | 77 | 79.0±1.8 |
| **MAIN** | 20167 | 19900±700 | 279 | 278.9±0.4 | 1023 | 1023±1 |
| **TOTAL** | 24545 | 24545 | 279 | 279 | 1024 | 1024 |
| **EDGE COMPONENTS** | **Internet** | **Internet randomized** | ***C. elegans*** | ***C.elegans* randomized** | ***E. coli*** | ***E. coli* randomized** |
| **ICE** | 20180 | 6700±400 | 2 | 0±1 | 16 | 15±5 |
| **ITF** | 10833 | 21500±1600 | 70 | 69±6 | 343 | 376±11 |
| **SCE** | 389 | 2150±160 | 1704 | 1693±11 | 3714 | 3540±20 |
| **OTF** | 226 | 950±110 | 172 | 150±6 | 160 | 189±6 |
| **OCE** | 12 | 140±40 | 7 | 0.9±1.1 | 2 | 3±2 |
| **MAIN** | 31640 | 31400±1700 | 1955 | 1912±6 | 4235 | 4123±11 |
| **TOTAL** | 45914 | 45914 | 1961 | 1961 | 4283 | 4283 |
