# Supplementary material for: Structural Efficiency of Percolated Landscapes in Flow Networks
Source: PLoS One. 2008 Nov 5;3(11):e3654. doi: 10.1371/journal.pone.0003654 (PMC2575234; doi:10.1371/journal.pone.0003654)
Supplement: Table S2 — Details of the interfaces and peripheral components of the Internet, C. elegans, and E. coli network representations. Decomposition of the interfaces and the peripheral node components. Values for the randomized versions of the networks are also provided (average standard deviation rounded off to the first significant figure). Edges at the interfaces and nodes at the components are separated into leafs (l) and non-leafs (nl), and nodes directly connected to the core (d = 1) are distinguished from those at larger distances (d>1). When present, bidirectional links are counted as single. (0.04 MB DOC) [file pone.0003654.s002.doc]

## TABLE S2. Details of the interfaces and peripheral components of the Internet, *C. elegans*, and *E. coli* network representations

Decomposition of the interfaces and the peripheral node components. Values for the randomized versions of the networks are also provided (average$\pm$standard deviation rounded off to the first significant figure). Edges at the interfaces and nodes at the components are separated into leafs (l) and non-leafs (nl), and nodes directly connected to the core (d=1) are distinguished from those at larger distances (d>1). When present, bidirectional links are counted as single.

| **IN COMPONENTS** | **Internet** | **Internet randomized** | ***C. elegans*** | ***C.elegans* randomized** | ***E. coli*** | ***E. coli* randomized** |
| --- | --- | --- | --- | --- | --- | --- |
| **IN d=1** | 8166 | 15800±800 | 12 | 11±1 | 125 | 126±4 |
| **IN d>1** | 11894 | 3130±180 | 0 | 0.0±0.1 | 7 | 0.0±0.7 |
| **IN l** | 6488 | 13900±800 | 11 | 10.8±0.4 | 113 | 113±1 |
| **IN nl** | 13572 | 5060±170 | 1 | 1±1 | 19 | 13±4 |
| **ITF l** | 7933 | 18200±1300 | 64 | 65±3 | 311 | 333±6 |
| **ITF nl** | 2900 | 3300±200 | 6 | 3±6 | 32 | 43±13 |
| **OUT COMPONENTS** | **Internet** | **Internet randomized** | ***C. elegans*** | ***C.elegans* randomized** | ***E. coli*** | ***E. coli* randomized** |
| **OUT d=1** | 16 | 110±20 | 30 | 27±1 | 77 | 78.8±1.7 |
| **OUT d>1** | 1 | 10±6 | 0 | 0.0±0.1 | 0 | 0.0±0.4 |
| **OUT l** | 12 | 21±2 | 26 | 26.0±0.2 | 76 | 76±1 |
| **OUT nl** | 5 | 100±20 | 4 | 0.8±0.9 | 1 | 2.6±1.8 |
| **OTF l** | 218 | 740±30 | 140 | 145±3 | 159 | 183±5 |
| **OTF nl** | 8 | 220±110 | 32 | 5±7 | 1 | 6±5 |
