# Supplementary material for: Structural Efficiency of Percolated Landscapes in Flow Networks
Source: PLoS One. 2008 Nov 5;3(11):e3654. doi: 10.1371/journal.pone.0003654 (PMC2575234; doi:10.1371/journal.pone.0003654)
Supplement: Table S3 — Distribution of C. elegans neurons by function type among the different node components. To check that our production of the semidirected network is consistent and meaningful we computed the distribution of neurons by function type among the different node components. C. elegans neuronal functional organization is similar to most other metazoans and basically consists of sensory neurons (se), interneurons (in), and motor neurons (mo). We follow the classification in [1], which separates these cells into the three main groups and their crossings. Our statistical analysis reveals a structured overlap between the node components and the functional organization of neurons by type. Interestingly and according to rational expectations, the IN is mainly formed by sensory neurons while the OUT primarily consists of motor neurons, apart from interneurons present in both. Neurons in the SCC span all the classes with similar proportions. (0.03 MB DOC) [file pone.0003654.s003.doc]

## TABLE S3. Distribution of *C. elegans* neurons by function type among the different node components

To check that our production of the semidirected network is consistent and meaningful we computed the distribution of neurons by function type among the different node components. *C. elegans* neuronal functional organization is similar to most other metazoans and basically consists of sensory neurons (se), interneurons (in), and motor neurons (mo). We follow the classification in [1], which separates these cells into the three main groups and their crossings. Our statistical analysis reveals a structured overlap between the node components and the functional organization of neurons by type. Interestingly and according to rational expectations, the IN is mainly formed by sensory neurons while the OUT primarily consists of motor neurons, apart from interneurons present in both. Neurons in the SCC span all the classes with similar proportions.

| **COMPONENT** | **se** | **se-in** | **se-mo** | **in** | **in-se** | **in-mo** | **mo** | **mo-se** | **mo-in** | **TOTAL** |
| --- | --- | --- | --- | --- | --- | --- | --- | --- | --- | --- |
| **IN** | 8 | 0 | 0 | 3 | 0 | 1 | 0 | 0 | 0 | 12 |
| **SCC** | 63 | 3 | 6 | 65 | 4 | 6 | 66 | 4 | 20 | 237 |
| **OUT** | 0 | 0 | 0 | 10 | 0 | 0 | 18 | 0 | 2 | 30 |
| **TOTAL** | 71 | 3 | 6 | 78 | 4 | 7 | 84 | 4 | 22 | 279 |

[1] Oshio K, Iwasaki Y, Morita S, Osana Y, Gomi S, Akiyama E, Omata K, Oka K, Kawamura K (2003). In Technical Report of CCeP, Keio Future No. 3, Keio University.
